# Liver Biopsy Handling of Metabolic-Associated Fatty Liver Disease (MAFLD): the Children’s Hospital of Eastern Ontario grossing protocol

**DOI:** 10.1177/20420188241227766

**Published:** 2024-02-04

**Authors:** Consolato M. Sergi, Mohit Kehar, Carolina Jimenez-Rivera

**Affiliations:** Division of Anatomic Pathology, Children’s Hospital of Eastern Ontario, University of Ottawa, 401 Smyth Road Ottawa, Ottawa, ON K1H 8L1m, Canada; Department of Laboratory Medicine and Pathology, Stollery Children’s Hospital, University of Alberta Hospital, Edmonton, AB, Canada; Division of Gastroenterology, Hepatology and Nutrition, Children’s Hospital of Eastern Ontario, University of Ottawa, Ottawa, ON, Canada; Division of Gastroenterology, Hepatology and Nutrition, Children’s Hospital of Eastern Ontario, University of Ottawa, Ottawa, ON, Canada

**Keywords:** histology, liver, MAFLD, NAFLD, steatohepatitis

## Abstract

Metabolic-(non-alcoholic) associated fatty liver disease (MAFLD/NAFLD) has increasingly become a worldwide epidemic. It has been suggested that renaming NAFLD to MAFLD is critical in identifying patients with advanced fibrosis and poor cardiovascular outcomes. There are concerns that the progression to non-alcoholic steatohepatitis (NASH) may become a constant drive in the future healthcare of children and adolescents. There is a necessity to tackle the emerging risk factors for NASH-associated hepatocellular carcinoma (HCC). In this narrative review, we present the current protocol of liver biopsy separated between pre-analytical, analytical, and post-analytical handling. Genetic association investigations have identified single nucleotide polymorphisms implicated in the progression of MAFLD-HCC, many of which seem to belong to the lipid metabolism pathways. PNPLA3 rs738409 variant, TM6SF2 rs58542926 variant, MBOAT7 rs641738 variant, and GCKR variants seem to be significantly associated with NAFLD disease susceptibility. In disclosing the current comprehensive protocol performed at the Children’s Hospital of Eastern Ontario, Ottawa, ON, Canada, we support the most recent Kulkarni-Sarin’s pledge to rename NAFLD to MAFLD. Grossing of the liver biopsy is key to identifying histologic, immunophenotypical, and ultrastructure data and properly preserving tissue for molecular genomics data.

## Plain language summary

Handling a biopsy with fatty liver disease

Fatty liver disease is increasing worldwide. There is a necessity to tackle the emerging risk factors for the development of liver cancer following years of fatty liver disease. In this paper, we show our protocol at the Children’s Hospital of Eastern Ontario, University of Ottawa, Ontario, Canada.

## Introduction

The liver biopsy is a paramount procedure in internal medicine and gastroenterology, aiming to identify a broad spectrum of etiologies ranging from abnormal values of liver function tests to structural changes or injuries at the hepatocellular level.^1–5^ Clinical evaluation entails a range from asymptomatic individuals to acute liver failure prompting liver transplantation. In the most recent definition of acute hepatitis, the American Association for the Study of Liver Disease categorizes an acute increase of liver biochemical values within six months of onset in a patient apparently not showing a pre-existing liver disease, while if the threshold of 6 months is over, the definition of chronic hepatitis is in place. In alcoholic liver disease, the liver may show changes stretching from simple fatty change to cirrhosis. The most common manifestation of alcoholic liver disease, which is also the earliest, is macrovesicular fatty change, which may develop within approximately six days of alcohol consumption, and following alcohol abstinence, this change may persist up to 6 weeks.^6–8^ It has been observed that alcohol and drug use has increased during the pandemic and school closures.^9^ This data may be relevant for the next epidemiological data of juvenile MAFLD (metabolic dysfunction-associated fatty liver disease)/NAFLD (non-alcoholic fatty liver disease).

**Figure 1.**
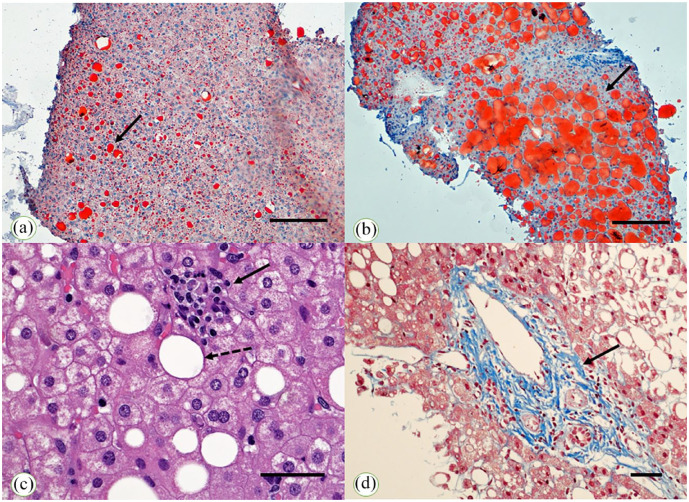
Upper microphotographs (a, b) show low power views of two patients (16-years-old male and 17-years-old female) with NAFLD/MAFLD and limited fat cell vacuolation (left, a) (the arrow points to the focal lipid infiltration of the hepatocytes) and massive fat cell vacuolation (right, b) (the arrow points to the diffuse lipid infiltration of the hepatocytes) arising from liver tissue kept frozen at −20°C and stained with ORO solution for fat (both microphotographs have been taken at 100× magnification; bar, 200 micrometers). The lower microphotographs (c, d) display a focus of lobular inflammation (lobulitis) on the left (c) (the solid arrow points to the focus of lobulitis, while the dashed arrow points to a hepatocyte with univacuolar change) in a 16-years-old male and expansion of the portal tract of the liver with fibrosis on the right (d) using Masson’s trichrome staining in a 17-years-old female (the solid arrow points to the expanded portal tract with fibrosis, which is blue-stained) (c, 400× magnification, bar, 25 micrometers; d, 200× magnification, bar, 10 micrometers). MAFLD/NAFLD, Metabolic-(non-alcoholic) associated fatty liver disease; ORO, Oil-red O.

The liver biopsy should be sent to the department of laboratory medicine and pathology fresh in a Petri dish with a few drops of saline, not using wet gauze to avoid artifacts. The number of cores may vary, and it depends on a number of factors, including the age of the patient, coagulation status, and a number of ancillary techniques that can be used to establish a pathogenetic link or identify prognostic factors. In our academic hospital, three fresh cores are commonly received. The potential occurrence of NASH and dysplastic or carcinomatous areas should prompt the pathologist to embed and freeze one core or part of one core in OCT (optimal cutting temperature) compound, which is a watery compound blend of glycols (polyethylene glycol and polyvinyl alcohol among other reactive and non-reactive ingredients) and resins. OCT provides a convenient cryo embedding matrix designed for sectioning using a cryostat of tissue specimens at temperatures of −10°C or below. Importantly, OCT does not leave any residue on slides during the staining procedure. It eliminates undesirable background staining.^10^ Although Cryo-Gel™ has been advocated for some investigations, OCT is used worldwide. The quality of DNA and RNA is good in liver tissue, and no significant differences have been observed between the distinct embedding compounds despite initial studies pointing out interference in the amplification by polymerase chain reaction.^10^ In case proteomic studies are considered and envisioned, OCT compound is better to be substituted by Cryo-Gel.^10^ Steatohepatitis is considered the triad of fatty change, Mallory hyaline, and inflammation is encountered histologically.^11,12^ Alternatively, a tetrad or a pentad have been indicated incorporating the presence of hepatocyte ballooning with or without fibrosis.^13,14^ In Figure 1, several aspects of fatty vacuolation, inflammation, and fibrosis are depicted. The presence of Mallory hyaline and fatty change has been associated with poor survival in patients with liver cirrhosis, including patients with cirrhosis operated on for relief of portal hypertension.^13^ If the inflammation and fatty change are often centrilobular in alcoholic hepatitis, these changes are less typically associated with a centrilobular distribution in non-alcoholic steatohepatitis (NASH). Non-alcoholic fatty liver disease (NAFLD), which is also currently, although not univocally termed MAFLD, is constituted by an aberrant accumulation of fat in hepatocytes owing to a metabolic derangement.^15^ Liver biopsy is still needed and has aimed to give a comprehensive picture of the liver disease of the child. Most often, pediatric NAFLD has a different type of histology compared to adult Zone 1/3 involvement commonly seen in adults. In addition, ballooning and Mallory’s bodies in children especially younger are not often seen as seen in adults.^13^

It seems crucial to spend some time exploring the NAFLD/MAFLD diatribe. Apart from the pandemic, the last three years have shown some divergence in the terminology of fatty liver disease. The NAFLD labeling and its definition have well-known limitations for both adult and pediatric populations.^16^ Alcohol consumption is usually not a concern in children and is not often verifiable in adults. This challenging definition has triggered a consensus to relabel and redefine NAFLD to MAFLD. An international panel proposed using MAFLD.^16^ The new MAFLD criteria provide pediatricians with a transparent scaffold for diagnosing the disease, its risk stratification, and new improved clinical care. The definition may be considered valid across the lifespan. Three issues have been considered, and they raised some concerns. At first, the selection of experts in the consensus panels was not completely transparent. Secondly, a further concern regarded the quantity and quality of the evidence proposed by the consensus panels. Finally, there was limited or poor input to the decisions of the consensus panel from the clinical and academic.^17^ Finally, a global multistakeholder agreement came into action to placate the diatribes eventually.^18^

This manuscript aims to elucidate the approach to liver biopsy to allocate and process liver biopsies optimally in the MAFLD setting and give a simple outline of the epigenomics investigations currently in place in Ottawa, ON, Canada. Standard procedures for liver biopsy handling and processing have been established in several organizations, and guidelines have been collected.^19–24^ We will divide the handling and processing into three sections, pre-analytical, analytical, and post-analytical. The pre-analytical handling entails taking the biopsy and transferring the material to the department of laboratory medicine and pathology. In the analytical handling, several tasks include dividing the sample, preserving the tissue, cutting the sample, staining the sample, reporting the finding, establishing the diagnosis, and sign-out the report. The post-analytical phase includes sending the reports to the physicians and/or incorporating into a laboratory information system (LIS, e.g. Epic Systems Corp. (EPIC) is an American privately held healthcare software company),^25^ interpretation of the report, clinical-pathological correlation, multidisciplinary team meetings, and liver conferences.

**Figure 2.**
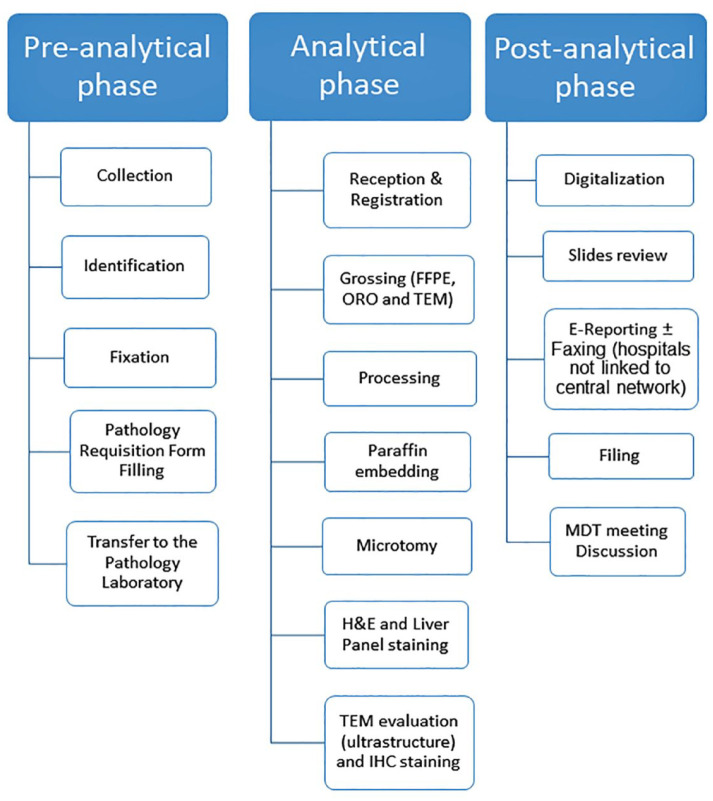
Flow Chart of the main steps of the pre-analytical, analytical, and post-analytical section of the liver tissue biopsy handling aimed to receive a diagnosis and prognostic score for clinical purposes.

Most recently, there has been some debate on converting the labeling NAFLD to MAFLD to account for factors of metabolism potentially influencing patients’ outcomes.^26^ In a systematic review and meta-analysis of over 10 million individuals, Chan *et al.* reported a 39% prevalence of MAFLD, of which 5% were labeled “lean” and 30% were labeled “non-obese.”^27^ In this journal, Kulkarni and Sarin discussed the alcohol consumption effects on fatty liver disease and the advantages and disadvantages in renaming NAFLD.^28^ In this narrative review, we present the protocol used at the Children’s Hospital of Eastern Ontario, University of Ottawa, in handling the liver biopsy of children with liver function test abnormalities, separating the pre-analytical handling from the analytical and post-analytical handling (Figure 2). In the pre-analytical phase, there are personnel responsible for specimen collection, matching two or three identifiers for patients, and filling out the requisition form with clinical information for the pathology laboratory. Most of the tissue is fixed in formaldehyde, but some portions of the tissue or some cores in some hospitals are preserved for cryostatic procedures, freezing, or processed for ultrastructural examination (Glutaraldehyde is usually used for transmission electron microscopy). An ideal scenario may involve a 3D barcode as used in some hospitals. The analytical phase includes reception and registration of the sample. The tissue is grossed by an experienced pathology assistant or the pathologist himself/herself. The pathologist is responsible to train correctly the personnel involved in the procedure after the tissue specimen is obtained as well as his or her personnel in grossing the liver biopsy. Formalin-fixation and paraffin embedding is the routine procedure to obtain tissue blocks and gather unstained tissue sections to be stained for hematoxylin and eosin and special stains (trichrome stain for collagen, reticulin stain to highlight reticulin fibers, which are constituted of type 3 collagen and are helpful to evaluate the lobular architecture, Perl’s iron stain for detecting iron, periodic acid Schiff stain with and without diastase digestion to detect undigested material (e.g. alpha-1-antitrypsin globules, basement membrane, intra-macrophagic debris, and fungal microorganisms) and glycogen, rhodanine stain for copper-binding proteins). The Oil red O staining requires unstained sections cut using cryo-static procedures. The pathologist should also train the transmission electron microscope (TEM) personnel in screening the fields of semi-thin sections for ultrastructural evaluation. In some cases, immunohistochemical stains are performed for specific cases (e.g. keratins 7 and 19 for biliary epithelium and bile duct changes, hepatitis B virus, hepatitis C virus, cytomegalovirus, parvovirus B19, and herpes simplex virus).^29^ The post-analytical phase includes digitalizing of the slides using a slide scanner, the review of the slides with or without fellows or residents, signing-out of the electronic reports using an electronic signature, and faxing the report to hospitals or clinicians not associated with the pathology laboratory network. Finally, the slides are filed, and a multi-disciplinary team meeting is set-up in 1–2 weeks’ time following the results of the ultrastructural examination are available.

## Liver biopsy pre-analytical handling

A histopathological investigation is required when data for diagnosis, management, therapy, or prognostication is unavailable from non-invasive techniques. Following institutional review board approval for ethics and written consent collection for clinical studies, a liver biopsy is indicated for research purposes to delineate categories and validate prognosis factors. However, in most of our patients, there is a clinical indication, and liver biopsy is part of the standard of care. The parents or guardians must sign a written consent. It should be obtained before the biopsy. The information provided to the parents or guardians should include the benefits and risks of the procedure, illustrate alternatives where appropriate, and indicate follow-up arrangements as post-handling procedures. It is our practice to provide verbal and written information in the official languages (English and French) with an optional native language if a translator is available. The format needs to be understandable by the patient, particularly if older than 12 years of age, and, where appropriate, their caretakers. It is recommended that plenty of time is given to the caretakers to assimilate the information provided. A healthcare practitioner who is acquainted with the techniques and has enough time for the patient or parents/guardians to ask any question should give it a few days before the biopsy is performed. Moreover, consent should be confirmed closely prior to the medical biopsy. Risks associated with a percutaneous liver biopsy include bleeding, organ perforation, infection, sepsis, and death. Risk factors for bleeding from percutaneous biopsy include comorbidities and abnormal coagulation values. It is controversially discussed, but there is little convincing evidence that operator status and number of passes pointedly increase the risk of bleeding. Mortality associated with biopsy is less than one in 1:10,000.^30^ Since the hematological parameters in several patients with liver disease are abnormal, with disturbance of both thrombolysis and coagulation, the traditional measures of platelet count and prothrombin time do not allow a confident consideration for the decision to carry on or not the invasive procedure and need to be adequately explained to the parents/guardians. In non-lesional biopsies, in patients with liver disease, a transjugular route should be used if the international normalized ratio is greater than 1.5. Since most liver biopsies are done by interventional radiologists, it is important to underline to the parents and guardians that this aspect has been associated with a reduced rate of complications and increase in adequate samples.^30,31^ It is useful and wise to obtain percutaneous liver biopsy under imaging guidance or ultrasound guidance. Generally, automated cutting-type special needles and that core biopsy needles (full) are routinely used. A 16 G needle should be applied for the percutaneous approach. However, an 18 G needle is opted for a solid lesion to access by percutaneous path. It is suggested that the length of the sample be at least 20 mm. Having a liver biopsy is not an easy task for both patient and family, and physician. We found out that communication overhauls fine print consent details and biopsy information. A communication between the family/patient and the pathologist with the clinician is crucial for the optimal management of patients.^32^ Percutaneous liver biopsy is the most common type of biopsy, and an ultrasound scanner or CT scan (computed tomography scan) may guide the clinician obtaining the biopsy for greater accuracy. In some cases, a blind liver biopsy is performed and takes place where the biopsy is done without imaging guidance. A blind liver biopsy should not be carried out without latest imaging of the liver. A transvenous/transjugular biopsy is performed in patients who have troubles with blood clotting, and the risk of bleeding cannot be minimized. Other types of liver biopsy comprise transfemoral, in which the femoral vein acts as access point, endoscopic ultrasound-guided liver biopsy, in which it is done endoscopically in the duodenum, and, finally, laparoscopically through the abdomen, either during surgery or through a laparoscope. If a biopsy is undertaken solely or partly for research purposes, full information to the patients must be provided. The procedure needs to be approved by the appropriate ethics committee in alignment with hospital regulations and patient’s safety guidelines. It is advisable that for non-urgent biopsies, the period between medical request and liver biopsy should be less than four weeks, regardless of which type of procedure is adopted, as quality standards.

The minimal size of the cores is variable from institution to institution. We suggest that a minimum of six portal tracts need to be present in the sample to be considered adequate for diagnosis. If the percentage of periportal or centrilobular involvement in a biopsy is used to determine the severity of a focal lesion, a small biopsy sample size will lead to considerable misevaluation of the liver disease. Thus, the size and quality of the tissue specimens are paramount for the quality of the service provided.

## Liver biopsy analytical handling

**Figure 3.**
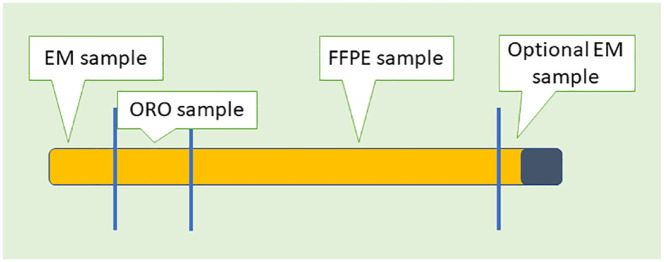
Schematic approach to a liver biopsy with a sample reserved for FFPE, a sample dedicated to ORO staining (special stain) for cryostat cutting and staining for fat cell quantification, and one or two samples for ultrastructural examination (EM). EM, electron microscopy; FFPE, formalin-fixation, paraffin embedding; ORO, oil-red O.

A dissecting microscope can be used to recognize cortical parenchyma, or in the absence of any visual guidance, the pathologist or a senior technologist can remove 1-mm cubes from each end of each core and place them in glutaraldehyde for ultrastructural examination using a TEM (Figure 3). Three cores are received in our institution, and the two additional cores are embedded in paraffin after formalin fixation for histology. The remaining core can be subsequently divided into two pieces, placing the larger of the core, about two-thirds of the core length, in fixative for light microscopy [formaldehyde, PBS (phosphate-buffered saline)-buffered at 10%], while one-third of the core length goes to an OCT-chuck (chuck using optimal cutting temperature compound) for frozen sections, which are immediately stained for Oil Red O (ORO) for assessment of the lipid accumulation. In conventional cryostating procedures, tissue is embedded by placing it face up on a cryostate tissue holder and covered with an embedding medium (OCT). The tissue holder is commonly labeled as “chuck” and it is then set upon a freezing temperature bar. Allocation should be carried out on a clean surface (such as a Petri dish or a wax cutting board) using a sharp blade to avoid crushing parts of the biopsy samples. We advise having one or two additional liver biopsies, which will be untouched fixed in a container with formaldehyde, PBS-buffered at 4–10% (pH 7.2–7.4) for light microscopy. This will be a second block and an optional third block will be used if a third block is received. The OCT-tissue should be kept frozen at −80°C for further metabolic studies if requested.

Forceps should not be used to avoid crushed artifacts, and it is paramount to avoid touching the tissue with a fixative-tainted scalpel or razor to avoid false-negative ORO results. ORO is a lysochrome or fat-soluble diazo dye. It is commonly used for staining of neutral triglycerides and lipids on frozen tissue sections. Occasionally, it can be used to stain some lipoproteins on paraffin sections. In our experience, the allocation of small pieces for TEM, which can be stored and processed later, provides the best opportunity for the diagnosis of inborn storage disorders (e.g. lysosomal storage disorders). Although Zenker fixative allows for the acquisition of optimal morphologic details, formalin fixation allows for much greater immunohistochemistry ancillary testing. We do not routinely perform immunofluorescence, but the core fragment in OCT can be used for this purpose if requested. Tissue for light microscopy is processed, dehydrated, and embedded in a paraffin block. Multiple serial sections at 2.5–3 cm thickness are cut and stained using an automatic stainer. Usual stains include hematoxylin and eosin, periodic acid-Schiff, after diastase treatment (PASD), Masson trichrome (alternatives are Sirius red, Ladewig, etc.), silver methenamine (Jones). We allow additional unstained slides to be cut to allow additional special studies as needed. We have 3–5 hours of processing, sectioning, and staining time as typically needed to generate tissue slides for light microscopy, according to a shortened protocol if needed. Immunohistochemistry is usually done to stain for the biliary type of (cyto-) keratins, 7 and 19.^33,34^ In addition, we stain using a polyclonal antibody against the carcinoembryonic antigen, which magnificently highlights the bile canaliculi in both humans and animal models.^4,35–37^

The ultrastructural procedure has been described in detail.^38^ The EM tissue specimen needs to be fixed in 2% or 3% glutaraldehyde). Substantially, the EM tissue is processed and embedded in a plastic, hard medium. Scout sections are stained with toluidine blue to identify the specific area to be cut for thin sections to be placed on a grid for the ultrastructural examination. In our center, two working days are typically needed to process the specimen and produce thin sections for ultrastructural examination. We and others found that microwave processing can accelerate polymerization and cut down the processing time, but morphology may be suboptimal for both kidney and liver tissue specimens.^39^ Salvage techniques may be used in case not enough tissue is obtained for a specific procedure. Salvage light microscopy can start from the frozen tissue core. Thus, the remaining frozen tissue after ORO staining may be retrieved. It can be fixed in buffered formalin 10% and, ultimately, processed for light microscopy. Freezing artifacts are common, and there is some loss of morphologic detail. Nevertheless, salvage light microscopy from frozen tissue can provide invaluable information when the original light microscopy material is inadequate. Salvage electron microscopy from formalin-fixed and paraffin-embedded (FFPE) tissue can be done, but artifacts are common and need to be cautiously separated by findings. Some membranes are abnormally thin due to artifacts owing to such processing, precluding accurate measurements. Salvage TEM from frozen tissue has major artifacts and is currently not recommended.

With regard to medical liver biopsies, the tissue should be received intact in a Petri dish and be constituted by three cores or two cores occasionally if the third core is difficult to obtain. All cores should be of sufficient size and able to show the lobular architecture of the liver. We recommend at least six well-formed portal tracts with the portal artery, portal vein, and interlobular bile duct triad. Portal tracts and hepatic veins are usually about 800 μm apart. Therefore, it is better advisable that a biopsy has a diameter of 0.8–1 mm. Such an aim can be reached using a 16 G needle. Although in adults, more than ten portal tracts are considered a minimum number to have a comprehensive examination of the liver, six portal tracts are the suggested number in pediatric patients in our opinion. Colloredo *et al.* showed that the proportion of biopsies exhibiting a minimum of bridging fibrosis was decreased from 41% in liver tissue, which was 3000 μm (micrometers) × 1400 μm (micrometers) to about 20% if the tissue was as wide but 1000 μm (1 mm) long, or 20–30 mm (2–3 cm) long but 1000 μm (1 mm) wide.^40^

The use of a second and, potentially, third core is needed for the accomplishment of several studies and clinical trials granted that an ethics committee approval was received for biorepositories.^41^ Some concern is commonly shared that more than one liver pass for medical reasons may increase the complication rate, but in our experience, there is no clear evidence on this point. We suggest that a third or a second pass should be carried out if the first liver biopsy core is less than 2000 μm long. We also suggest that the second and third cores may be necessary for biochemical, genetic, and ultrastructural investigations avoiding jeopardizing the histologic interpretation, which remains the first goal of the liver biopsy. If there is a clinical differential suspicion of Wilson’s disease, a fragment of the core of liver tissue should be given for copper assessment, which is usually handled by the department of chemical pathology. The consideration of Wilson’s disease must be handled with caution. Wilson’s disease liver tissue can disclose steatosis. Thus, such a consideration remains critical in pediatric departments. The tissue for copper analysis should be enfolded in moistened sterile paper. It should not be floating in formalin fixative, formalin solution, alcohol, or saline. Similarly, the same process is required for iron content, although it is rarely required nowadays. If the clinical diagnosis includes MAFLD/NAFLD or NASH as well as acute fatty liver of pregnancy, the liver cores should be sent fresh to be one of them or part of them frozen section for fat.

We use ORO stain for 1 mm^3^ liver tissue, which should not be subcapsular. Microvesicular steatosis in such conditions (MAFLD/NAFLD or NASH as well as acute fatty liver of pregnancy AFLP) may disguise some challenges in formalin-fixed paraffin-embedded sections. AFLP is a clinical emergency (obstetrics), which carries a high rate of morbidity and mortality for both mother and fetus. Fresh tissue samples for metabolic disease may also be stored from pediatric patients, and liaising with the department of genetics is critical to confirm the amount of tissue, which is needed for genetic investigations.

Communication remains critical not only for autopsy reports but also for liver biopsy tissue, and we adopt the in-person discussion of the liver tissue at the microscope station.^32,42^ It is critical that the clinician provides the pathologist with all clinical information for the purpose to enable a correct interpretation of the liver biopsy and provide a clinically useful histopathologic report. All results of biochemistry, immunology, and imaging should be reviewed in team and discussed with the appropriate physician management team. Where appropriate, proper scoring systems should be routinely used.

The final report of a liver biopsy should include information on the adequacy of the specimen (# of the portal tracts and triads), a description of the morphological changes in a systematic fashion for each of the compartments of interest (portal, periportal or zone 1, central and pericentral or zone 3, and midzonal or zone 2), the results of the immunohistochemical studies, and the results of the ultrastructural study.

The pathology report should discuss the biopsy size and adequacy, length of the single cores, number of an approximate number of portal tracts evaluated for all tissue submitted for histopathologic evaluation. In addition, the lobular architecture needs to be assessed with regard to the presence and severity of fibrosis, the type of fibrosis (periportal, pericentral, perisinusoidal), the distortion of liver *muralium* or hepatocyte plates, the ducto-vascular relationships, and parenchymal nodularity. A description of the histological changes and histochemical phenotype needs to be added. With regard to the MAFLD/NAFLD or NASH as well as acute fatty liver of pregnancy, an attempt should be made to give an indication of the main pattern of morphological change discovered in the liver. Grading and staging are also critical, and we adopt the Clinical Research Network system for scoring the activity. In Table 1 is shown the activity score of NAFLD. It includes the scores of fatty changes, lobulitis, and hepatocytic ballooning (range: 0–8). Details are present in the original publication of Brunt and Tiniakos.^43^

**Table 1.** NASH activity according to clinical research network system.

Fatty change	Lobulitis	Hepatocyte ballooning
0: <5%	0: None	0: None
1: 5–33%	1: <2	1: Few ballooned cells
2: 34–66%	2: 2–4	2: Many ballooned cells
3: > 66%	3: >4	

The activity score of NAFLD includes the scores of fatty changes, lobulitis, and hepatocytic ballooning (range: 0–8). Details are in the original publication of Brunt and Tiniakos.^43^

NAFLD, non-alcoholic associated fatty liver disease; NASH, non-alcoholic steatohepatitis.

It is also critical to compare the current liver tissue with any previous liver biopsies. This aspect is especially important if the purpose of the biopsy is to investigate disease progression or response to treatment. In the comment, a clinicopathological consideration should be added. This should also add any discussion with the responsible clinician. A succinct, single- or double-line summary to conclude the report is also important. We advise using coding for the purposes of archiving and billing (e.g. ICD-# or SNOMED-CT code). The laboratory information system EPIC seems highly relevant for clinical and academic purposes.^25^ The Synchronized Nomenclature in Medicine is a recognized structural medical vocabulary for use in an electronic clinical record.^25^ In addition, a record (including names or initials) of any intradepartmental consultation and outside referral for a second opinion should be part of a supplementary report if there is no change in a diagnosis, which otherwise should trigger an amendment of the report.

## Liver biopsy post-analytical handling

Epigenomics studies may be critical for the full interpretation of microvesicular steatosis, and biorepositories are critical. Single Cell Gene Sequencing following laser capture microdissection is carried out to target the questions of the molecular phenomics of the cells associated with the inflammation and identification of Mallory hyaline. Single Cell Gene Expression Flex lets us identify areas that need to be carefully evaluated.^44^ Single cell gene expression simplifies experimental logistics by enabling storage and transport without losing data quality, augments single cell profiling to include FFPE tissues from clinical trials or other translational studies, and group and multiplex tissue samples to maximize efficiency and limit variability. A number of SNPs are being established in our institution. In our institution, we target the role of m6A-associated genes as diagnostic and prognostic biomarkers.^44^

NAFLD has a variable natural history and heterogeneous progression of liver damage. Advances in human genetics present new opportunities to address the unmet need for NASH therapeutics based on an improved understanding of the multifactorial pathogenesis of NASH and the complex interaction between genetic and environmental risk factors. Genetics and its interplay with epigenetic and environmental factors (diet/exercise) could determine the variation in the disease progression.^45^ The strong genetic component was based on the observation that there is a significant racial and ethnic difference in the prevalence of NAFLD and clustering amongst the family members.^46–49^ Hispanic children have the highest prevalence (11.8%) of NAFLD, and black children (1.5%) have the lowest in an autopsy-based study in which diagnosis was based on liver histopathology.^50^ These differences have also been seen in adulthood.^51^ The clustering of NAFLD within families had been evaluated, showing adjusted heritability estimates of 1 (with 0 being no heritability and 1 representing a trait that is completely heritable).^52^ In a landmark genome-wide association study, the rs738409 C > G single nucleotide polymorphism (SNP) encoding a common missense variant of patatin-like phospholipase domain-containing 3 (*PNPLA3*) gene was associated with NASH. SNPs in other genes, including *TM6SF2* (Trans-Membrane 6 Super-Family member 2) rs58542926 on 19p13.11 (Modulate hepatic VLDL secretion (Very-Low-Density Lipoprotein), *GCKR* (Glucokinase Regulator) rs780094 on 2p23.3 (Modulate hepatic lipogenesis), *MBOAT7* (Membrane Bound O-Acyl Transferase domain containing 7) rs641738 on 19q13.42 (Remodeling of phosphatidylinositol), and *HSD17B13* (Hydroxy-Steroid 17-beta dehydrogenase 13) rs72613567: TA on 4q22.1 (Retinol dehydrogenase activity) have been associated with NAFLD development and/or progression. There is rapid evolution in this space over past few years with more new SNPs being detected which are associated with NAFLD onset and progression.^26,45,50–53^ The *PNPLA3* rs738409 variant seems to be paramountly associated with NAFLD disease susceptibility among several populations across continents. However, gene variants like *TM6SF2* rs58542926, *MBOAT7* rs641738 and *GCKR* gene variants do not appear also ethnically selective. In NASH, circulating miR-122 was reported to be upregulated and suggested as a potential prognosis biomarker.^46,48,54^ There are emerging data highlighting that *PNPLA3, MBOAT7*, and *STAT3* affect the occurrence and development of MAFLD. The *PNPLA3* gene mutation is associated with an increase of liver transaminase activity. This data can obviously promote the progression of liver disease to pronounced steatosis and liver fibrosis. MBOAT7 is used to catalyze the desaturation of the second acyl chain of phospholipids and transfer polyunsaturated fatty acids, while STAT3 plays a key role in the process of leptin resistance. High levels of leptin may be related to the increase of NAFLD/MASLD. *TM6SF2* and *GATAD2A* genetic variants have also been detected in some populations.^55^ *STAT3* gene polymorphisms need to be explored in the future in different populations and liver disease states. The activation of STAT3 may lead to cytokine signal inhibitor-3 (SOCS-3) playing a feedback inhibitory role by weakening the leptin receptor signal transduction.^55^ With regard to the progression of NASH to cancer with or without cardiovascular disease, ethnicity seems to play an important role among other risk factors.^56,57^ SNPs in several genes have been linked with a notably increase of the risk to develop hepatocellular carcinoma (HCC).^56^ Variants in *PNPLA3, TM6SF2, MBOAT*, and *GCKR* seem to be highly relevant. Gellert-Kristensen *et al.* indicated that a genetic risk score comprising three common variants in the genes *PNPLA3, TM6SF2*, and *HSD17B13* (rs72613567) was associated with up to 12-fold higher risk of liver cirrhosis, and up to 29-fold higher risk of liver cancer.^58^

Bisulfite conversion is a method used to distinguish methylated from unmethylated cytosines in genomic DNA at single base resolution. DNA is first denatured and then treated with sodium bisulfite. Sodium bisulfite selectively changes unmethylated cytosines into uracil through deamination while leaving methylated cytosines both 5-methylcytosine and 5-hydroxymethylcytosine) unchanged.^44,59,60^ Large-scale methylome profiling may involve either methylation microarray or methylation sequencing with next-generation sequencing (NGS).^61–64^ The starting input material for both methods involves 100–200 ng and the specimen can be fresh, frozen or FFPE, although the use of FFPE is suboptimal comparing with either fresh or frozen tissue. Clinical applications of gene expression or methylome profiling may be relevant for prognostic and/or predictive scopes with clinical trials as aim, and artificial intelligence tools may be key in the future to discern prognostic biomarkers (Table 2). The MGMT (*O*^6^-methylguanosine-DNA methyltransferase) promoter hypermethylation is also key in our forthcoming studies. MGMT is a DNA repair enzyme, and the gene is located on 10q26.3.^65^ Epigenetic silencing of the MGMT gene by promoter methylation has been associated with decreased production of the enzyme, which may compromise this DNA repair mechanism and may predict response to alkylating agent therapy (such as temozolomide). In addition, MGMT gene promoter methylation status is a prognostic biomarker in pediatric and adult patients with glioblastoma. Therefore, testing for MGMT gene promoter methylation has become an important assay for identifying patients who will have a better overall prognosis and may respond better to alkylating agents.

**Table 2.** Current methodologies.

Parameter	Gene expression profiling	Methylome profiling
Input material	RNA	DNA
Strategy	**Direct** – GE levels	**Indirect** – GE regulation
Specimen	Fresh/Frozen tissue >>> FFPE	FFT or FFPE
Data Reproducibility	Variable with tendency to low consistency	Variable with tendency to high consistency
Data analysis	Scores and classification	Scores and classification
Data interpretation	Human-based or AI-based	Human-based or AI-based

The role of the gut or bile microbiome is still obscure and needs additional investigation in both human^66–68^ and animal model studies.^69,70^ There is always almost some frozen tissue. Thus, in case there is some funding, we could start some epigenomics. As you may know, we have been involved in a group studying the gut microbiome and evolution toward cancer. The gut microbiome may alter the NAFLD progression. Nothing is known in children. It would be good if you could add the microbiome of the patient in the follow-up. Extend works on the microbiome and NAFLD with previous publications. We agree that the proportion of “fatty liver disease” may increment by enlarging the criteria of MAFLD renaming NAFLD.^28^ We fully support the concept that criteria need to be simple with the aim of educating primary care physicians and pediatricians who can recognize individuals who may be at higher risk of advanced fibrosis and poor cardiovascular outcome. Recently, nanopore sequencing has been key for several tasks in molecular genomics.^44^ Nanopore sequencing gives researchers several advantages for whole-genome sequencing, including easier assembly with long sequencing reads, meaning fewer fragments to assemble, faster results with real-time analysis, and long reading, which can extend entire structure variants in single reads. These qualities may be critical in evaluating NAFLD/MAFLD in the future.

In conclusion, for more than two centuries following the introduction of microscopy for liver tissue assessment, the microscope plays a critical role in managing NAFLD/MAFLD and NASH. The introduction of single-cell sequencing techniques and epigenomic investigations are promising to unveil many mysteries in liver pathology, and the introduction of machine learning algorithms with human-based methodology will be a key component of the pathology report shortly.

## Declarations

**Ethics approval and consent to participate:** Not applicable.

**Consent for publication:** Not applicable.

**Availability of data and materials:** All PDFs and data are publicly available.
